# A comparison of COVID-19 epidemiological indicators in Sweden, Norway, Denmark, and Finland

**DOI:** 10.1177/1403494820980264

**Published:** 2021-01-07

**Authors:** Erica A. Yarmol-Matusiak, Lauren E. Cipriano, Saverio Stranges

**Affiliations:** 1 Ivey Business School, Western University, London, ON, Canada; 2 Department of Epidemiology and Biostatistics, Schulich School of Medicine & Dentistry, Western University, London, ON, Canada; 3 Department of Family Medicine, Schulich School of Medicine & Dentistry, Western University, London, ON, Canada; 4 Department of Population Health, Luxembourg Institute of Health, Strassen, Luxembourg

**Keywords:** COVID-19, Nordic, policy analysis, COVID-19 mortality, infectious disease transmission, infectious disease prevention, public health strategy

## Abstract

*Aims:* To compare the early impact of COVID-19 infections and mortality from February to July 2020 across the Nordic nations of Sweden, Norway, Denmark, and Finland through available public data sources and conduct a descriptive analysis of the potential factors that drove different epidemiological outcomes, with a focus on Sweden’s response. *Methods:* COVID-19 cases, deaths, tests, case age distribution, and the difference between 2020 all-cause mortality and the average mortality of the previous 5 years were compared across nations. Patterns in cell phone mobility data, testing strategies, and seniors’ care home deaths were also compared. Data for each nation were based on publicly available sources as of July 31, 2020. *Results:* Compared with its Nordic peers, Sweden had a higher incidence rate across all ages, a higher COVID-19-related death rate only partially explained by population demographics, a higher death rate in seniors’ care, and higher all-cause mortality. Sweden had approximately half as much mobility change as its Nordic neighbours until April and followed similar rates as its neighbours from April to July. Denmark led its Nordic peers in testing rates, while Sweden had the highest cumulative test-positivity rate continuously from mid-March. Conclusions: COVID-19 pushed Sweden’s health system to its capacity, exposed systemic weaknesses in the seniors’ care system, and revealed challenges with implementing effective contact tracing and testing strategies while experiencing a high case burden. Looser government restrictions at the beginning of the outbreak are likely to have played a role in the impact of COVID-19 in Sweden. In an effort to improve epidemic control, Sweden has increased testing rates, implemented more restrictive prevention measures, and increased their intensive care unit bed capacity.

## Background

Policy decisions at the beginning of the COVID-19 epidemic were made under tremendous uncertainty. Variation in government and public health policies during the COVID-19 pandemic has resulted in different national and community trajectories of COVID-19. Many countries have implemented community-centered public health policies such as social distancing protocols, rapid testing, and contact tracing to test, track, isolate, and provide medical treatment to individuals with COVID-19. The intensity of implementation—both from the perspective of government recommendations compared with mandates as well as the adherence to recommendations by the public—have resulted in different outcomes across countries with similar population demographics, economic systems, and health care infrastructures.

Sweden’s public health responses to COVID-19 were less restrictive and were instituted more slowly than neighbouring nations, which spurred substantial controversy [1, 2]. The four Nordic nations of Sweden, Norway, Denmark, and Finland have similar demographic and economic profiles as well as comparable health care systems and public health infrastructures, though they maintain unique national identities as well as differences in population density [3], geography, culture, and governmental organization. These differences are likely to have influenced health policy decision-making during COVID-19, however, broader similarities between the Nordic countries enable useful comparisons to determine the relative impacts of the differences in public health responses to the COVID-19 pandemic.

With cases in late January and February associated directly with travel from affected regions, community spread had begun in all four countries by early March [3]. Norway, Denmark, and Finland mandated the closing of workplaces and schools as well as many other services between March 10 and March 16; Sweden recommended comparable closing measures between March 21 and 25 [4]. On several other containment measures, Sweden introduced less strict, voluntary measures several days or weeks later than its neighbours [4, 5] (Supplemental Figure 1). All nations adapted early public health responses to evolving evidence about the infectiousness and the natural history of COVID-19 including adverse clinical sequelae such as severe morbidity or mortality in vulnerable populations.

Our objective was to conduct a descriptive analysis of publicly available epidemiological indicators across Nordic countries to determine the early impact of COVID-19 infections and mortality from February to July 2020 and explore potential factors driving differences between countries, with a focus on Sweden’s response. We compared the current epidemiologic data up to July 31, 2020 across the four Nordic countries including COVID-19 cases, deaths, tests, and case age distribution. All-cause mortality per 1000 population in 2020 was compared with an average from 2015 to 2019. This comparison helps explore the impact of excess all-cause mortality given the potential underestimation of COVID-19 cases and mortality during a public health crisis. Finally, we explored sources of differences in outcomes across the countries including comparative analyses of cell phone mobility data, testing strategies, and seniors’ care home deaths as potential drivers of outcome differences between the four nations.

## Methods

Data were derived from a variety of online sources up to July 31, 2020. Detailed references for each data source are presented in Supplemental Table 1. Cumulative cases, cumulative deaths, age distribution data, and daily cases were from Statista [6]. Cumulative tests per 1000 population, daily testing data per 1000 population, and test-positivity rates were from Our World in Data [7]. Mobility data were extracted from the Google Community Mobility Report, with region-specific baseline values established using a median of the corresponding day of the week from the period between January 3 and February 6, 2020 [8]. Weekly all-cause death data were sourced from Norway, Denmark, and Finland’s national statistics agencies; daily all-cause death data were sourced from Statistics Sweden and aggregated into weekly rates. We also present all-cause mortality data for Stockholm county to investigate excess mortality in Sweden’s epicenter region. All-cause mortality and cumulative deaths are presented up to July 5 because of the 1-week lag in reporting and the relatively high rate of data correction that occurs over the most recent 3 weeks. COVID-19-related deaths in long-term care were sourced from a variety of reports from national social services and health agencies. To ensure comparability across countries, case, COVID-specific death, and all-cause mortality counts were converted to rates per 1000 population using country populations for each year from 2015 to 2020.

## Results

### Incidence rate and testing

**Figure 1.**
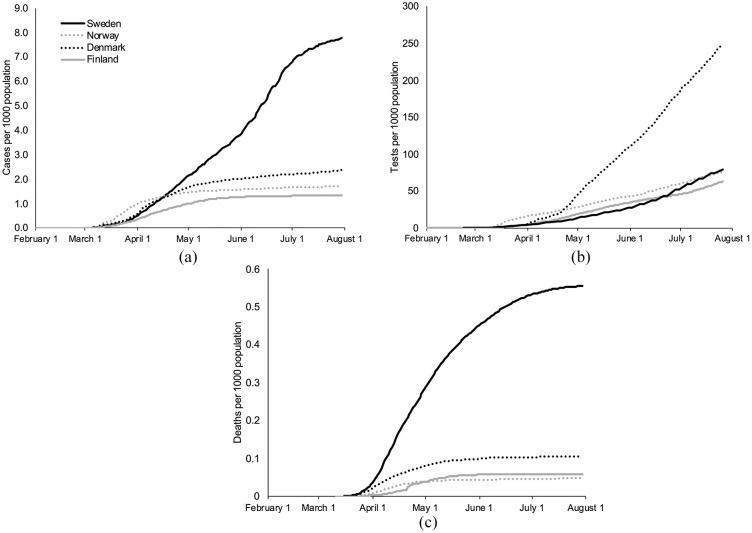
(a) Cumulative cases of COVID-19 per 1000 population; (b) cumulative tests for COVID-19 per 1000 population, seven day moving average; (c) cumulative deaths from COVID-19 per 1000 population. Case and test data are included until July 31; death data are included until July 5.

Daily COVID-19 incidence varied across Nordic countries (Supplemental Figure 2(a)), first increasing in Norway followed by increasing incidence rates in Denmark, Sweden, and Finland. After more than 4 months of active cases, compared with its Nordic neighbours, Sweden had the highest number of COVID-19 cases per 1000 population (Figure 1A): 7.8 per 1000 population in Sweden compared with 1.7, 2.4, and 1.4 per 1000 population in Norway, Denmark, and Finland, respectively.

Testing also varied across Nordic countries and may partially explain differences in case detection. Denmark has maintained a daily testing rate 3 to 4 times its neighbours since mid-April (Figure 1(b), Supplemental Figure 2(b)). The cumulative testing rate per 1000 population was 79.7 tests (Sweden), 76.5 tests (Norway), 259.7 tests (Denmark) and 64.8 tests (Finland). The cumulative test-positivity rate was 9.7% (Sweden), 2.3% (Norway), 1.0% (Denmark) and 2.1% (Finland). The test-positivity rate has changed dramatically over the course of the pandemic for all countries with peaks in March for Norway (9.5%), Denmark (17.5%), and Finland (11.9%), and an April peak in Sweden (19.1%). Current rates are below 1% in Norway, Denmark, and Finland and at 2.9% in Sweden (Supplemental Figure 2(c)).

### Mortality

Higher case incidence corresponds to overall higher COVID-19 specific mortality rates. Sweden, with a COVID-19 attributed death rate of 0.54 per 1000 population as of July 5, has a higher death rate compared with its neighbours (Figure 1(c)): 11.5× compared with Norway (0.05 deaths per 1000 population), 5.1× compared with Denmark (0.10 deaths per 1000 population), and 9.1× compared with Finland (0.06 deaths per 1000 population).

**Figure 2.**
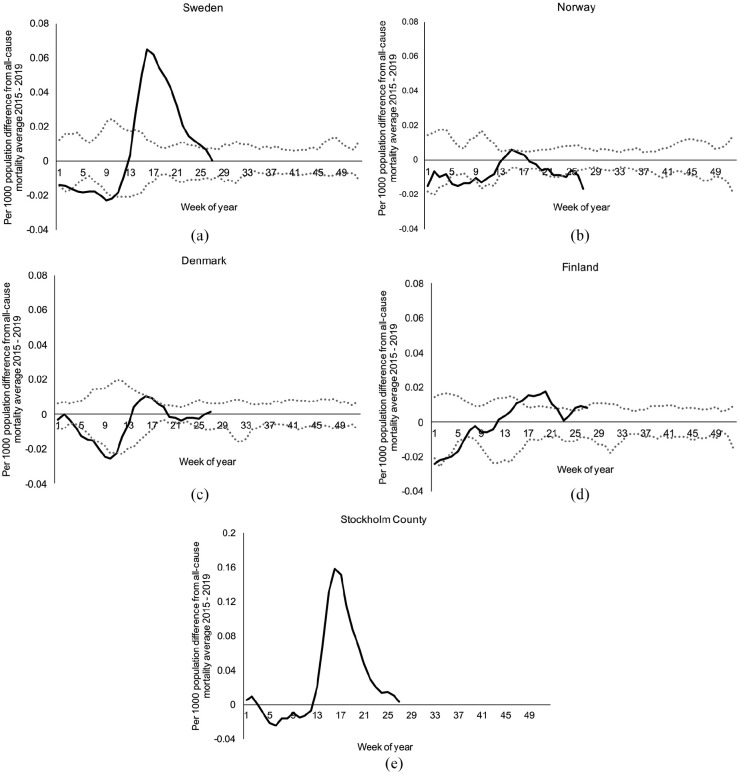
Absolute difference between the all-cause mortality per 1000 population in 2020 and the average all-cause mortality per 1000 population in 2015 to 2019 for each week: (a) Sweden; (b) Norway; (c) Denmark; (d) Finland; and (e) Stockholm County in Sweden. Solid lines represent 2020 weekly all-cause mortality per 1000 population. Dotted lines represent the minimum and maximum observed weekly all-cause mortality per 1000 population from 2015 to 2019. All lines represent a 3-week moving average. Data are included until July 5.

In Figure 2, we present the absolute difference between the weekly all-cause mortality rate in 2020 and the average weekly all-cause mortality rate for the previous 5 years (2015 to 2019) for each Nordic nation as well as for Stockholm county in Sweden. All four Nordic countries and Stockholm county started the first 2 months of the year near the minimum observed weekly mortality rate of the last 5 years. Beginning in March, all-cause mortality rates increased to rates more consistent with the highest observed weekly mortality rate over the last 5 years. Compared with the expected weekly all-cause mortality rate, the absolute number of excess deaths in Sweden from week 13 to week 27 in 2020 was 5388 deaths (5% higher than expected) (Figure 2(a)). In contrast, Norway and Denmark had decreases in cumulative mortality of 5%, 3%, and Finland has had a 0.7% increase thus far in 2020. The 5% increase in total cumulative mortality in Sweden is a national average aggregating relatively harder hit regions with regions that had very few cases. Specifically, Stockholm county represents 23% of the population of Sweden, but accounts for 41% of all COVID-19 deaths. Focusing on all-cause mortality (Figure 2(e)), Stockholm county experienced a 22% increase in all-cause mortality in 2020 compared with the average of the previous 5 years. Stockholm county also has a more rapidly decreasing slope than Sweden as a whole, indicating that the virus continued to affect other sub-regions even after Stockholm was able to implement additional control measures.

### Age distribution of infections and deaths in seniors’ care

**Figure 3.**
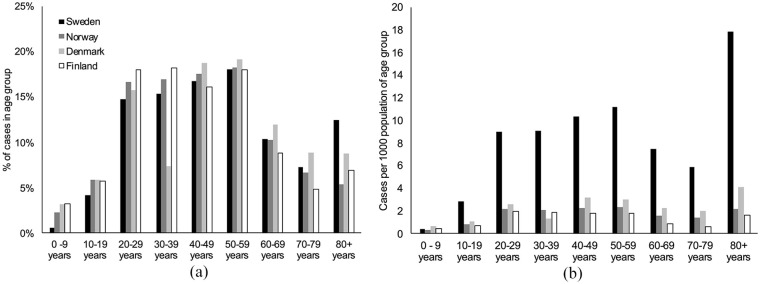
(a) Distribution of COVID-19 cases by age; (b) age-specific incidence of COVID-19 per 1000 population. Data are included until July 31.

Sweden’s higher COVID-19 mortality burden may partially be explained by its age distribution of the population or the age distribution of infections: 13% of the detected COVID-19 cases in Sweden were in people aged ⩾80 years, compared with between 5% and 9% for its neighbours (Figure 3(a)). This is consistent with different distributions of age-specific incidence rates: among people aged ⩾80 years, the age-specific incidence rate in Sweden was 6.8 times greater than the average age-specific incidence rates of Norway, Denmark, and Finland for people aged ⩾ 80 years (versus 4 to 5 times greater among 20 to 80-year-olds) (Figure 3(b)). Seniors’ care homes have accounted for a sizable proportion of all COVID-19 attributed deaths within each nation: 45% (Sweden), 60% (Norway), 35% (Denmark), and 44% (Finland). Among nations in the Organisation for Economic Co-operation and Development (OECD), the average fraction of deaths in seniors’ care homes is 42% (Supplemental Figure 4).

### Mobility data

**Figure 4.**
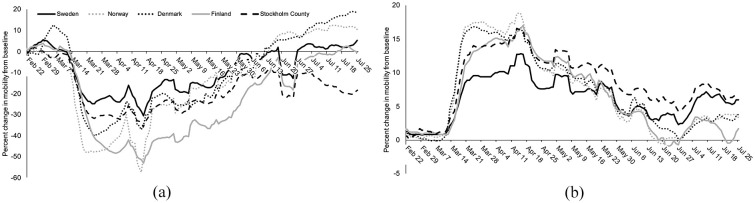
(a) Retail and recreation mobility, percent change from baseline; (b) residential mobility, percent change from baseline. All lines represent a 7-day moving average. Baseline values are region-specific and were established using a median of the corresponding day of the week from the period between January 3 and February 6, 2020. Note: Public holidays occurred for all four countries on April 10, April 13, and May 21; on May 1 for Norway/Sweden/Finland; on June 19 for Sweden/Finland; on April 9 and June 1 for Norway/Denmark; and May 8, May 22, and June 5 for Denmark. Data are included until July 31 and are from the Google Community Mobility Report.

Following public health policies and recommendations, large retail and recreation mobility decreases and residential time increases occurred in mid-March (Figure 4). However, Sweden had approximately half as much mobility change (about –20%) as its Nordic neighbours (–40% to –50%) with regards to retail and recreation mobility in late March to mid April. There was also less change in Sweden’s residential mobility compared with its Nordic neighbours over the late March to mid-May period. Stockholm county shows trends closer to its Nordic neighbours than Sweden as a whole. By mid-May, mobility data indicate that the people of Sweden were staying at home at a similar rate as their Nordic neighbours and continue at this rate into early July even as their Nordic neighbours return to baseline residential time at home. Additional mobility data for workplaces, transit, parks, and grocery & pharmacy is shown in Supplemental Figure 3.

## Discussion

Compared with its Nordic peers, Sweden experienced higher incidence rates across all ages and a higher COVID-19-related death rate. Though all four nations experienced the impact of COVID-19 in seniors’ care homes, Sweden experienced the highest death rate per 1000 population in this care setting. During the COVID-19 pandemic, cumulative all-cause mortality decreased in Norway and Denmark, but increased in Sweden, with the Stockholm county epicenter responsible for a large portion of the mortality increase. In addition, Sweden’s low rate of testing and high cumulative test-positivity rate suggest high rates of case under-detection in the community over the course of the outbreak. Our work provides an early comparative analysis of COVID-19 epidemiological indicators and the potential effects of COVID-19 on all-cause mortality in Nordic countries, exploring the potential sources of differential outcomes such as testing practices and population behaviour in response to recommendations.

### Early epidemic cases linked to travellers

International flight connectivity has largely been responsible for spreading cases to locations around the world, later igniting community transmission and fostering larger outbreaks. Finland had the first case in the Nordic region, detected on January 29 in a tourist from China [9]. Community transmission did not occur, and Finland reacted quickly starting to ban travel from high-risk regions earlier than neighbours (Supplemental Figure 1), which, in tandem with a comparatively high rate of testing, may have contributed to limiting their burden of overall COVID-19 infections and mortality from February to July. Return from winter holidays in central Europe, especially from the northern Italy epicenter, may have driven early initial cases upwards. Though all the Nordic countries were affected, Norway had high connectivity to this region [10], which may have contributed to a COVID-19 increase in cases per capita in March prior to its neighbours (Figure 1). Among other measures, Norway responded with travel restrictions and rapid testing increases (Supplemental Figure 2) which are likely to have helped detect and prevent the spread of cases.

### Role of government regulations as an early response measure

Using variation in policies and publicly announced cases and mortality data across regions in the United States, Goolsbee and Syverson attributed a large portion of decline in mobility to voluntary stay-at-home decisions based on people’s fear of COVID-19 deaths in their region, with legal restrictions accounting for only ~12% of the mobility decrease [11]. Consistent with this, Andersen et al. found similar reductions in consumer spending in Sweden and Denmark (25% c.f. 29%) despite substantially greater government restraints on economic activity in Denmark [12]. Focusing only on Stockholm county, mobility data indicate a population response to increasing case and mortality counts preceding increasing government restrictions. However, the national analysis we present provides counterevidence as well: Norway, Denmark, and Finland enacted strict and early government regulations (Supplemental Figure 1) prior to observing high case counts or COVID-19 associated deaths and, ultimately, maintained all-cause mortality rates below or equal to the average for the last 5 years. Despite increasing cases and connectivity to global news, mobility data show that the population-based response in Sweden overall was not as strong as its neighbours and did not intensify to their level as the situation worsened. Given that the median time from first symptoms of COVID-19 to death is 14 days (95% CI: 6–41)[13], government restrictions can be implemented as a form of early response measure to encourage a stronger shift in population behaviour before a rise in cases and deaths occurs [14, 15].

### Variations in testing strategy

All four Nordic countries started with similar strategies of testing suspected cases and travelers with symptoms. As COVID-19 cases increased, testing strategies shifted to include high-risk groups and individuals with severe respiratory symptoms [16–18]. Initially, in all nations, medical referrals were needed to receive a test, which may have presented a barrier to access. Denmark was first to implement a broader testing strategy, opening testing up to people with mild symptoms in April and to all adults without referrals in May [19]. Denmark’s high absolute testing rate led to a relatively low test-positivity rate as more mildly symptomatic and asymptomatic cases were detected, helping use testing as a strategy to prevent transmission. (Supplemental Figure 2). Using testing as a central mechanism to lift confinement restrictions was first introduced successfully in several East Asian countries [14, 20]. South Korea implemented a nationwide mass scale-up of testing, contact tracing, and isolation of individuals, which are likely to have contributed to their ability to avoid instituting stay-at home orders [14]. Vietnam attributes its ability to effectively manage the pandemic through testing only high-risk and suspected cases, in combination with extensive contact tracing and strict quarantines of affected areas. Sweden did not begin expanding testing until late May and did not achieve test-positivity rates below the World Health Organization recommended threshold of 5% until July, which may have contributed to increasing the magnitude and the duration of the COVID-19 burden [17].

### Sub-regional variations in COVID-19 burden and excess mortality in epicenter regions

Sub-regional geography can contribute to substantial within-country variation in terms of COVID-19 cases and deaths. National averages tend to obscure both extremes of COVID-19’s outsized impact on higher density urban centers as well as the much smaller regional outbreaks in less connected cities. The Nordic nations have similar levels of urbanization and fractions of total population in their capitals (Supplemental Figure 5). However, Denmark’s national population density is 5 to 10 times that of Sweden, Norway, and Finland, and Copenhagen is the densest capital city, which is likely to have had an impact on their approach to managing the pandemic [21]. In Sweden, 55% of COVID-19 cases were in Stockholm and Västra Götaland municipalities by the end of July, and the majority of other regions had case incidence rates similar to other Nordic countries. Excess mortality in Stockholm county mirrors trends seen around the globe with internationally connected, high density metropolitan areas being initial epicenters of virus transmission. Examples include case fatality rates of 18.5% in Lombardy, Italy [15] and sevenfold increases in all-cause mortality in New York City at the peak of the pandemic [22]. Major urban centers are also points of connectivity to other cities creating a source of cases for neighbouring domestic communities. Despite efforts to control the spread in major hubs, other low population density regions may experience a smaller rise in cases simultaneously or after outbreaks in large cities.

### Impact of health systems at capacity

Overwhelmed health systems can contribute to increased COVID-19 transmission because capacity constraints may result in restrictive prioritization systems for testing, overwhelmed resources for contact tracing, and delayed testing results (further reducing the benefits of contact tracing and case isolation). Overwhelmed systems can also lead to higher case fatality rates because patients cannot receive medically indicated care when demand for physical resources (e.g., mechanical ventilators, dialysis equipment, personal protective equipment (PPE)) or human resources (e.g., respiratory therapists, nurses) exceeds capacity. Both of these pressures may have contributed to severe outcomes in Sweden; we observed that Sweden had the highest test-positivity rate and the second lowest per-capita rate of testing. Further, prior to the COVID-19 pandemic, Sweden also had the lowest number of intensive care unit (ICU) beds per capita among the Nordic countries [23]. Sweden’s peak number of ICU beds occupied with COVID-19 cases was 558 beds on April 25, indicating their health system was running above its pre-pandemic capacity of 526 ICU beds in 2019 [24, 25]. Sweden rapidly scaled up its surge ICU capacity throughout April from 743 beds to 1131 beds [26], enabling care for more individuals. However, utilization of surge resources often means seconding staff and other resources from their usual roles, teams working under stress [27], and transporting critically ill patients significant distances to hospitals with equipment and human resource capacity [28].

### Systemic challenges in seniors’ care homes

Sweden experienced disproportionate incidence among the very elderly and nearly half of all COVID-attributed deaths occurred in seniors’ care homes [1, 29]. Pierre discusses the institutional arrangements and challenges of the Swedish seniors’ care system that are likely to have contributed towards this failure: decentralized leadership often run at municipal level; privatization; underfunding of public care homes; highly mobile employees who work at multiple facilities; and workers lacking infectious disease training, equipment, and PPE [30, 31]. COVID-19 has similarly had an impact on seniors’ care facilities in many other developed countries including Canada, Spain, and France [32]. Sweden has recently allocated USD 220 million for training and resources for the seniors’ care sector [33], and reform of these kinds of systemic challenges in long-term care has become a priority in several other OECD nations.

### Uncertainty and adaptability in public health decision making

Early public health response decisions were made under tremendous scientific uncertainty around the infectiousness and natural history of COVID-19, while trying to find a balance between protecting the population, avoiding overwhelming the health system, protecting individual freedoms, and maintaining a functioning employment-based and consumer-driven economic system. In retrospect, Sweden’s decisions underestimated the impact of asymptomatic transmission [30] and lacked foresight about how underlying challenges in the seniors’ care system would affect their support staff and elderly inhabitants. Sweden’s chief epidemiologist admitted that “if we were to encounter the same disease again…we would settle on doing something in between what Sweden did and what the rest of the world has done” [34] in an interview on June 3. When Sweden’s initial response resulted in the rapid spread of COVID-19, the Swedish population responded by reducing their contacts, and Sweden adapted with more restrictive measures, increasing their ICU bed capacity, and shifting their testing strategy. More recently, the nation has shown early signs of controlling the outbreak with new cases per day in the low hundreds (Supplemental Figure 2) and a return to national weekly all-cause mortality near the average of 2015 to 2019 (Figure 2).

Our understanding of the pathogen and policies that work to maintain healthy populations and resilient health systems have evolved; in hindsight, many other nations would likely have made different decisions to protect certain high-risk subgroups, put stricter controls on travel in certain regions, and allowed for certain parts of the economy or education systems to remain open. Sweden also stands as an example that initially deviated from stricter policy strategies adopted elsewhere but has been able to adapt in an agile manner and move towards control of COVID-19.

One challenge of drawing conclusions about public health during an ongoing pandemic is that we have limited scope of observation on the possible longer-term population health impacts. Johan Giesecke stated that it is too early to know if restrictive lockdowns will reduce mortality sustainably in the long run, and that countries may ultimately reach a similar mortality rate regardless of containment measures taken earlier in COVID-19 [35]. Different long-term outcomes may be determined by the effectiveness of efforts to support those economically harmed by COVID-19 and by strategies for disseminating information about less invasive prevention efforts (i.e., mask wearing), as well as efficient distribution of effective treatments and vaccines, once developed. By exposing shortfalls in existing institutions, COVID-19 may also act as a catalyst globally to spur investments in emergency preparedness, global public health, restructuring seniors care, and national healthcare systems.

### Limitations

Limitations of this study include delays in data reporting; varying definitions, reporting, and tracking guidelines; and country-specific differences in symptom guidance. We excluded the most recent 4 weeks of death data because the most recent 3 weeks are subject to data lags and updates. Data are updated at different frequencies between countries, and definitions for seniors’ care in reporting may also vary. Country-specific instructions to isolate for people presumed positive (vs. to get tested) may encourage certain self-isolating behaviour and affect case and test counts.

Types of data available in real time also limit the kinds of analyses that can be conducted. Limited availability of comparable sub-regional data about policies and outcomes for all regions within a nation makes it challenging to analytically explore within-country heterogeneity of public health decisions. In addition, detailed information is not available for a variety of other forces that may contribute to the outcomes we have investigated, including messaging from international media, local media reach, and individual community level factors.

## Conclusions

Over the first 6 months of the current pandemic, Sweden experienced higher COVID-19 case rates, death rates, and higher all-cause mortality (especially in the Stockholm county epicenter) than its Nordic peer countries. COVID-19 pushed Sweden’s health system to its capacity, exposed systemic vulnerabilities in the seniors’ care system, and revealed challenges with limited testing implementation. Choosing fewer and less intense government restrictions at the beginning of the outbreak likely played a role in the impact of COVID-19 in Sweden, although a connection to international news and rising fear of cases and deaths may have driven Swedish citizens to voluntarily shift to stay-at-home behaviour and reduce their spending. In spite of initial challenges, Sweden was able to adapt with more restrictive mandates, increasing their ICU bed capacity, and shifting their testing strategy. Sweden may provide an example of how an adaptive test-and-learn strategy can be applied to public health decisions made in uncertain times.

Despite the large media coverage around Sweden’s COVID-19 response, there is little academic literature systematically comparing Nordic countries’ policy responses to COVID-19 and their impact on epidemiologic indicators. This work adds to a growing body of literature to understand potential factors that affect variation in country burdens of COVID-19, and may provide an informative comparison for additional research on policy responses in Nordic countries.

## Supplemental Material

sj-pdf-1-sjp-10.1177_1403494820980264.pdf – Supplemental material for A comparison of COVID-19 epidemiological indicators in Sweden, Norway, Denmark, and Finland Click here for additional data file. Supplemental material, sj-pdf-1-sjp-10.1177_1403494820980264.pdf for A comparison of COVID-19 epidemiological indicators in Sweden, Norway, Denmark, and Finland by ERICA A. YARMOL-MATUSIAK, LAUREN E. CIPRIANO and SAVERIO STRANGES in Scandinavian Journal of Public Health

## References

[1] Häkkinen L. Hallengren: Ett misslyckande att vi inte lyckats skydda våra äldre [Hallengren: A failure that we have not managed to protect our elderly], SVT Nyheter, www.svt.se/nyheter/inrikes/hallengren-ett-misslyckande-att-vi-inte-lyckats-skydda-vara-aldre (2020, accessed 14 July 2020).

[2] Tegnell A. Tegnell: Fler åtgärder hade behövts – Nyheter (Ekot) [Tegnell: More preventive measures were needed], Sveriges Radio, sverigesradio.se/sida/artikel.aspx?programid=83&artikel=7487188 (accessed 14 July 2020).

[3] Juranek S Zoutman F. The effect of social distancing measures on intensive care occupancy: evidence on COVID-19 in Scandinavia. SSRN Electron J. Epub ahead of print 22 April 2020. DOI: 10.2139/ssrn.3577213.

[4] Hale T Webster S Petherick A, et al Coronavirus Government Response Tracker, www.bsg.ox.ac.uk/research/research-projects/coronavirus-government-response-tracker (2020, accessed 31 July 2020).

[5] Paterlini M. ‘Closing borders is ridiculous’: the epidemiologist behind Sweden’s controversial coronavirus strategy. Nature, 21 4, www.nature.com/articles/d41586-020-01098-x (2020, accessed 14 July 2020). 10.1038/d41586-020-01098-x 32317784

[6]] Statista. Statista – The statistics portal for market data, market research and market studies, www.statista.com/ (accessed 31 July 2020).

[7]] Our World in Data. Coronavirus pandemic (COVID-19) – statistics and research, https://ourworldindata.org/coronavirus (accessed 31 July 2020).

[8] Google. COVID-19 community mobility reports, www.google.com/covid19/mobility/ (accessed 31 July 2020).

[9] Tiirinki H Tynkkynen LK Sovala M, et al COVID-19 pandemic in Finland – preliminary analysis on health system response and economic consequences. Heal Policy Technol. Epub ahead of print 2020. DOI: 10.1016/j.hlpt.2020.08.005. PMC7451008 32874860

[10] Brynildsrud O Eldholm V. High COVID-19 incidence among Norwegian travellers returned from Lombardy: implications for travel restrictions. Epub ahead of print 2020. DOI: 10.1101/2020.03.20.20038406.

[11] Goolsbee A Syverson C. Fear, Lockdown, and diversion: comparing drivers of pandemic economic decline 2020. Cambridge, MA: National Bureau of Economic Research Epub ahead of print June 2020. DOI: 10.3386/w27432. PMC7687454 33262548

[12] Andersen AL Hansen ET Johannesen N, et al Pandemic, shutdown and consumer spending: lessons from scandinavian policyresponses to COVID-19*, https://arxiv.org/pdf/2005.04630.pdf (2020, accessed 7 July 2020).

[13]] WangWTangJWeiF. Updated understanding of the outbreak of 2019 novel coronavirus (2019-nCoV) in Wuhan, China. J Med Virol 2020;92:441–7.10.1002/jmv.25689PMC716719231994742

[14] Shokoohi M Osooli M Stranges S. COVID-19 pandemic: what can the West learn from the East? Int J Heal policy Manag 2020;2020:1–3. 10.34172/ijhpm.2020.85 PMC7719217 32610736

[15] Pasquariello P Stranges S. Excess mortality from COVID-19: a commentary on the Italian experience. Int J Public Health 2020;65:529–31. 10.1007/s00038-020-01399-y 32468219

[16] Norwegian Institute of Public Health. Test criteria for coronavirus, www.fhi.no/en/op/novel-coronavirus-facts-advice/testing-and-follow-up/test-criteria-for-coronavirus/ (2020, accessed 14 July 2020).

[17] Edwards C. Sweden announces major overhaul of coronavirus testing strategy. The Local, 4 6, www.thelocal.se/20200604/sweden-announces-major-overhaul-of-coronavirus-testing-strategy (2020, accessed 14 July 2020).

[18] Finnish Institute for Health and Welfare. Coronavirus tests – when should you get tested?, https://thl.fi/en/web/infectious-diseases-and-vaccinations/what-s-new/coronavirus-covid-19-latest-updates/symptoms-and-treatment-coronavirus/coronavirus-tests (2020, accessed 14 July 2020).

[19] Sundheds-Aedreministeriet. Alle borgere får mulighed for at blive testet for COVID-19 [All citizens will have the opportunity to be tested for COVID-19], www.sum.dk/Aktuelt/Nyheder/Coronavirus/2020/Maj/Alle-borgere-faar-mulighed-for-at-blive-testet-for-COVID-19.aspx (2020, accessed 14 July 2020).

[20] Scarpetta S Pearson M Colombo F, et al Testing for COVID-19: A way to lift confinement restrictions, www.oecd.org/coronavirus/policy-responses/testing-for-covid-19-a-way-to-lift-confinement-restrictions-89756248/#section-d1e85 (2020, accessed 25 July 2020).

[21]] WetterlingA. Derfor håndterer Sverige og Danmark coronakrisen så forskelligt [Why Sweden and Denmark handle the coronavirus crisis so differently], www.dr.dk/nyheder/udland/derfor-haandterer-sverige-og-danmark-coronakrisen-saa-forskelligt (2020, accessed 7 October 2020).

[22] Weinberger DM Chen J Cohen T, et al Estimation of excess deaths associated with the COVID-19 pandemic in the United States, March to May 2020. JAMA Intern Med. Epub ahead of print 2020. DOI: 10.1001/jamainternmed.2020.3391. PMC7330834 32609310

[23] Rhodes A Ferdinande P Flaatten H, et al The variability of critical care bed numbers in Europe. Intensive Care Med 2012;38:1647–53. 10.1007/s00134-012-2627-8 22777516

[24] Intensivvårdsregistret S. COVID-19 in Swedish intensive care, www.icuregswe.org/en/data–results/covid-19-in-swedish-intensive-care/ (2020, accessed 14 July 2020).

[25] Atallah C. Läkare: Brist på intensivvårdsplatser i landet [Doctors: Lack of intensive care units in the nation], www.svt.se/nyheter/inrikes/lakare-brist-pa-intensivvardsplatser-i-landet (2020, accessed 14 July 2020).

[26] Hallengren L. Sweden’s response to COVID-19: April 23 WHO Briefing. Government Offices of Sweden, https://www.government.se/49960e/globalassets/government/dokument/socialdepartementet/powerpoint-presentation-sweden-ministry-of-health-23-april-who-briefing-pdf.pdf (2020, accessed 7 July 2020).

[27] Löfgren E. ‘The biggest challenge of our time’: How Sweden doubled intensive care capacity amid Covid-19 pandemic. The Local, 23 6, www.thelocal.com/20200623/how-sweden-doubled-intensive-care-capacity-to-treat-coronavirus-patients (2020, accessed 14 July 2020).

[28] Winkelmann J Scarpetti G Hernandez-Quevedo C, et al How do the worst-hit regions manage COVID-19 patients when they have no spare capacity left? COVID-19 Health System Response Monitor, https://analysis.covid19healthsystem.org/index.php/2020/04/24/how-do-the-worst-hit-regions-manage-covid-19-patients-when-they-have-no-spare-capacity-left/ (2020, accessed 29 July 2020).

[29] Ali S Asaria M Stranges S. COVID-19 and inequality: are we all in this together? Can J Public Heal 2020;111:415–16. 10.17269/s41997-020-00351-0 PMC7310590 32578185

[30] Pierre J. Nudges against pandemics: Sweden’s COVID-19 containment strategy in perspective. Policy Soc 2020;39:478–493. 10.1080/14494035.2020.1783787 PMC8754703 35039732

[31] Karlsson C-J. Sweden’s failure to protect its elderly population from coronavirus started long before the pandemic, https://foreignpolicy.com/2020/06/23/sweden-coronavirus-failure-anders-tegnell-started-long-before-the-pandemic/ (accessed 14 July 2020).

[32] Canadian Institute for Health Information. Pandemic experience in the long-term care sector. How does canada compare with other countries? https://www.cihi.ca/sites/default/files/document/covid-19-rapid-response-long-term-care-snapshot-en.pdf (2020, accessed 10 July 2020).

[33] Swedish Ministry of Finance. Nya åtgärder för att stärka äldreomsorgen och vården under coronakrisen [New preventive measures to strengthen social services and healthcare for the elderly during the corona crisis], www.regeringen.se/pressmeddelanden/2020/05/nya-atgarder-for-att-starka-aldreomsorgen-och-varden-under-coronakrisen/ (2020, accessed 25 July 2020).

[34] Gehrke L. Swedish epidemiologist admits to flaws in country’s coronavirus response, Politico, 25 11, www.politico.eu/article/swedish-epidemiologist-admits-to-flaws-in-countrys-coronavirus-response/ (2020, accessed 14 July 2020).

[35] Giesecke J. The invisible pandemic. Lancet 2020;395:e98. 10.1016/S0140-6736(20)31035-7 PMC7200128 32539940

