# Supplementary material for: A comparison of COVID-19 epidemiological indicators in Sweden, Norway, Denmark, and Finland
Source: Scand J Public Health. 2021 Jan 7;49(1):69–78. doi: 10.1177/1403494820980264 (PMC7797349; doi:10.1177/1403494820980264)
Supplement: sj-pdf-1-sjp-10.1177_1403494820980264.pdf – Supplemental material for A comparison of COVID-19 epidemiological indicators in Sweden, Norway, Denmark, and Finland [file sj-pdf-1-sjp-10.1177_1403494820980264.pdf]

A comparison of COVID-19 epidemiological indicators in Sweden, Norway, Denmark, and Finland

Erica A. Yarmol-Matusiak, Lauren E. Cipriano, Saverio Stranges

SUPPLEMENTAL MATERIAL

Supplementary Table 1: Detailed Data References

Data	Reference
**Cumulative cases**	
Sweden	Statista. Cumulative number of coronavirus (COVID-19) cases in Sweden since February 2020, https://www.statista.com/statistics/1102203/cumulative-coronavirus-cases-in-sweden/. (accessed 31 July 2020)
Norway	Statista. Cumulative number of coronavirus cases in Norway since February 2020, https://www.statista.com/statistics/1102249/cumulative-coronavirus-cases-in-norway/ (accessed 31 July 2020).
Denmark	Statista. Cumulative number of coronavirus (COVID-19) cases in Denmark since February 2020, https://www.statista.com/statistics/1102241/cumulative-coronavirus-cases-in-denmark/ (accessed 31 July 2020).
Finland	Statista. Cumulative number of coronavirus (COVID-19) cases in Finland since January 2020, https://www.statista.com/statistics/1102255/cumulative-coronavirus-cases-in-finland/ (accessed 31 July 2020).
**Cumulative tests**	
All countries	Our World in Data. Total COVID-19 tests per 1,000 people, https://ourworldindata.org/grapher/full-list-cumulative-total-tests-per-thousand (accessed 31 July 2020).
**Cumulative deaths**	
Sweden	Statista. Cumulative number of coronavirus (COVID-19) deaths in Sweden since March 11, 2020, https://www.statista.com/statistics/1105753/cumulative-coronavirus-deaths-in-sweden/ (accessed 31 July 2020).
Norway	Statista. Cumulative number of coronavirus (COVID-19) deaths in Norway since March 12, 2020, https://www.statista.com/statistics/1105635/cumulative-coronavirus-deaths-in-norway/ (accessed 31 July 2020).
Denmark	Statista. Cumulative number of coronavirus (COVID-19) deaths in Denmark since March 11, 2020, https://www.statista.com/statistics/1106692/cumulative-coronavirus-deaths-in-denmark/ (accessed 31 July 2020).
Finland	Statista. Cumulative number of coronavirus (COVID-19) deaths in Finland since March 20, 2020, https://www.statista.com/statistics/1107435/cumulative-coronavirus-deaths-in-finland/ (accessed 31 July 2020).
**All-cause mortality**	
Sweden	Statistics Sweden. Population statistics - Preliminary statistics on deaths (updated 2020-07-03), https://www.scb.se/en/finding-statistics/statistics-by-subject-area/population/population-composition/population-statistics/ (accessed 31 July 2020).
Norway	Statbank Norway. Deaths, by sex, age and week. Preliminary figures 2000 - 2020, https://www.ssb.no/en/statbank/table/07995/ (accessed 31 July 2020).
Denmark	Statistics Denmark. Deaths per week (experimental statistics) by region, sex and age, https://www.statbank.dk/dodc2 (accessed 31 July 2020).
Finland	Statistics Finland. Deaths by Area, Week, Age, Sex and Information, https://pxnet2.stat.fi/PXWeb/pxweb/en/Kokeelliset_tilastot/Kokeelliset_tilastot__vamuu_koke/statfin_vamuu_pxt_12ng.px (accessed 31 July 2020).
**Age distribution of cases**	
Sweden	Statista. Number of coronavirus (COVID-19) cases in Sweden in 2020, by age groups, https://www.statista.com/statistics/1107905/number-of-coronavirus-cases-in-sweden-by-age-groups/ (accessed 31 July 2020).
Norway	Statista. Number of coronavirus (COVID-19) cases in Norway in 2020, by age and gender, https://www.statista.com/statistics/1103986/number-of-coronavirus-covid-19-cases-in-norway-by-age-groups/ (accessed 31 July 2020).
Denmark	Statista. Number of coronavirus (COVID-19) cases in Denmark in 2020, by age and gender, https://www.statista.com/statistics/1103966/number-of-coronavirus-covid-19-cases-in-denmark-by-age-and-gender/ (accessed 31 July 2020).
Finland	Statista. Number of coronavirus (COVID-19) cases in Finland as of July 17, 2020, by age group, https://www.statista.com/statistics/1103926/number-of-coronavirus-cases-in-finland-by-age-group/ (accessed 31 July 2020).
**Daily cases**	
Sweden	Statista. Number of new coronavirus (COVID-19) cases in Sweden since February 2020, by date of report, https://www.statista.com/statistics/1102193/coronavirus-cases-development-in-sweden/ (accessed 31 July 2020).
Norway	Statista. Number of new coronavirus (COVID-19) cases in Norway since February 2020, by date of report, https://www.statista.com/statistics/1102246/coronavirus-cases-development-in-norway/ (accessed 31 July 2020).
Denmark	Statista. Number of new coronavirus (COVID-19) cases in Denmark since February 2020, by date of report, https://www.statista.com/statistics/1102237/coronavirus-cases-development-in-denmark/ (accessed 31 July 2020).
Finland	Statista. Number of new coronavirus (COVID-19) cases in Finland since January 2020, by date of report, https://www.statista.com/statistics/1102251/coronavirus-cases-development-in-finland/ (accessed 31 July 2020).
**Daily tests**	
All countries	Our World in Data. Coronavirus (COVID-19) Testing, https://ourworldindata.org/coronavirus-testing (accessed 31 July 2020).
**Daily test positivity rate**	
Sweden	Calculated via Our World in Data daily testing data and Statista daily case data, cited above in this table

Norway, Denmark, Finland	Our World in Data. The share of COVID-19 tests that are positive, https://ourworldindata.org/grapher/positive-rate-daily-smoothed?tab=chart&country=~SWE (2020, accessed 31 July 2020).
**Population and age-specific population**	
Sweden	Statistics Sweden. Mean population (by year of birth) by region, age and sex. Year 2006 - 2019, http://www.statistikdatabasen.scb.se/pxweb/en/ssd/START__BE__BE0101__BE0101D/MedelfolkFodelsear/ (accessed 19 July 2020).
Norway	Statistics Norway. Population, by sex and one-year age groups (M) 1986 - 2020. Statbank Norway, https://www.ssb.no/en/statbank/table/07459/ (accessed 19 July 2020).
Denmark	Statistics Denmark. Population figures from the censuses, https://www.statbank.dk/statbank5a/default.asp?w=1280 (accessed 19 July 2020).
Finland	Statistics Finland. Population and population by age group, https://www.stat.fi/tup/suoluk/suoluk_vaesto_en.html#Population and population projection by age group (accessed 19 July 2020).
**Changes in government policy**	
All countries	Hale T, Webster S, Petherick A, et al. Coronavirus Government Response Tracker, https://www.bsg.ox.ac.uk/research/research-projects/coronavirus-government-response-tracker (2020, accessed 31 July 2020)
**Public holidays**	
All countries	Nordea Bank. Nordea Bank Holidays, https://www.nordea.com/en/about-nordea/contact/bank-holidays/ (accessed 14 July 2020).
**Seniors care deaths**	
Sweden	Socialstyrelsen. Statistics on the number of deaths in covid-19, https://www.socialstyrelsen.se/statistik-och-data/statistik/statistik-om-covid-19/statistik-over-antal-avlidna-i-covid-19/ (2020, accessed 31 July 2020).
Norway	Folkehelseinstituttet. Covid-19 Ukesrapport-uke 30. (2020, accessed 31 July 2020).
Denmark	Statens Serum Institut. Overvågning af COVID-19, https://www.ssi.dk/sygdomme-beredskab-og-forskning/sygdomsovervaagning/c/covid19-overvaagning (2020, accessed 31 July 2020).
Finland	Forma L, Aaltonen M, Pulkki J. COVID-19 and clients of long-term care in Finland-impact and measures to control the virus, https://ltccovid.org/wp-content/uploads/2020/06/ltccovid-country-reports_Finland_120620.pdf (12 June 2020, accessed 31 July 2020).
**Urbanization (%)**	
All countries	World Bank. Urban population (% of total population) - Norway, Sweden, Denmark, Finland, https://data.worldbank.org/indicator/SP.URB.TOTL.IN.ZS?end=2019&locations=NO-SE-DK-FI&start=2019&view=bar (2018, accessed 7 October 2020)
**Population density**	
All countries	World Bank. Population density (people per sq. km of land area)- Norway, Sweden, Denmark, Finland, https://data.worldbank.org/indicator/EN.POP.DNST?locations=NO-SE-DK-FI (2018, accessed 7 October 2020)
**Capital city populations**	
Sweden	Statistics Sweden. 50 largest municipalities, by population, https://www.scb.se/en/finding-statistics/statistics-by-subject-area/population/population-composition/population-statistics/pong/tables-and-graphs/rank-lists-municipalities/swedens-50-largest-municipalities-2019/ (2019, accessed 12 October 2020)
Norway	Statistics Norway. Population. Statbank Norway, https://www.ssb.no/en/befolkning, (accessed 12 October 2020)
Denmark	Statistics Denmark. Population 1 January by municipality and time, https://www.statbank.dk/BY2, (2020,accessed 12 October 2020)
Finland	Statistics Finland. Population by municipality-based units, https://www.stat.fi/org/avoindata/paikkatietoaineistot/vaesto_tilastointialueittain_en.html (2020, accessed 12 October 2020)
**Capital city land areas**	
Sweden	Statistics Sweden, Land and water area 1 January by region and type of area (no update after 2011). Year 2000 – 2011, https://www.statistikdatabasen.scb.se/pxweb/en/ssd/START__MI__MI0802/Areal/ (2020, accessed 12 October 2020)
Norway	Statistics Norway. Population and land area in urban settlements, https://www.ssb.no/en/beftett/(2020, accessed 12 October 2020)
Denmark	New World Encyclopedia. Copenhagen. https://www.newworldencyclopedia.org/entry/copenhagen (2017, accessed 12 October 2020)
Finland	Statistics Finland. NLS National Land Survey of Finland - Official Municipality Land Area for 2014, https://www.maanmittauslaitos.fi/sites/maanmittauslaitos.fi/files/old/alat_2014.pdf (2014, accessed 12 October 2020)

Supplementary Figure 1: Graphical representation of strictness of Nordic countries' eight containment and closure measures by date. Data is included until July 31 and is from the Oxford Coronavirus Government Response Tracker.

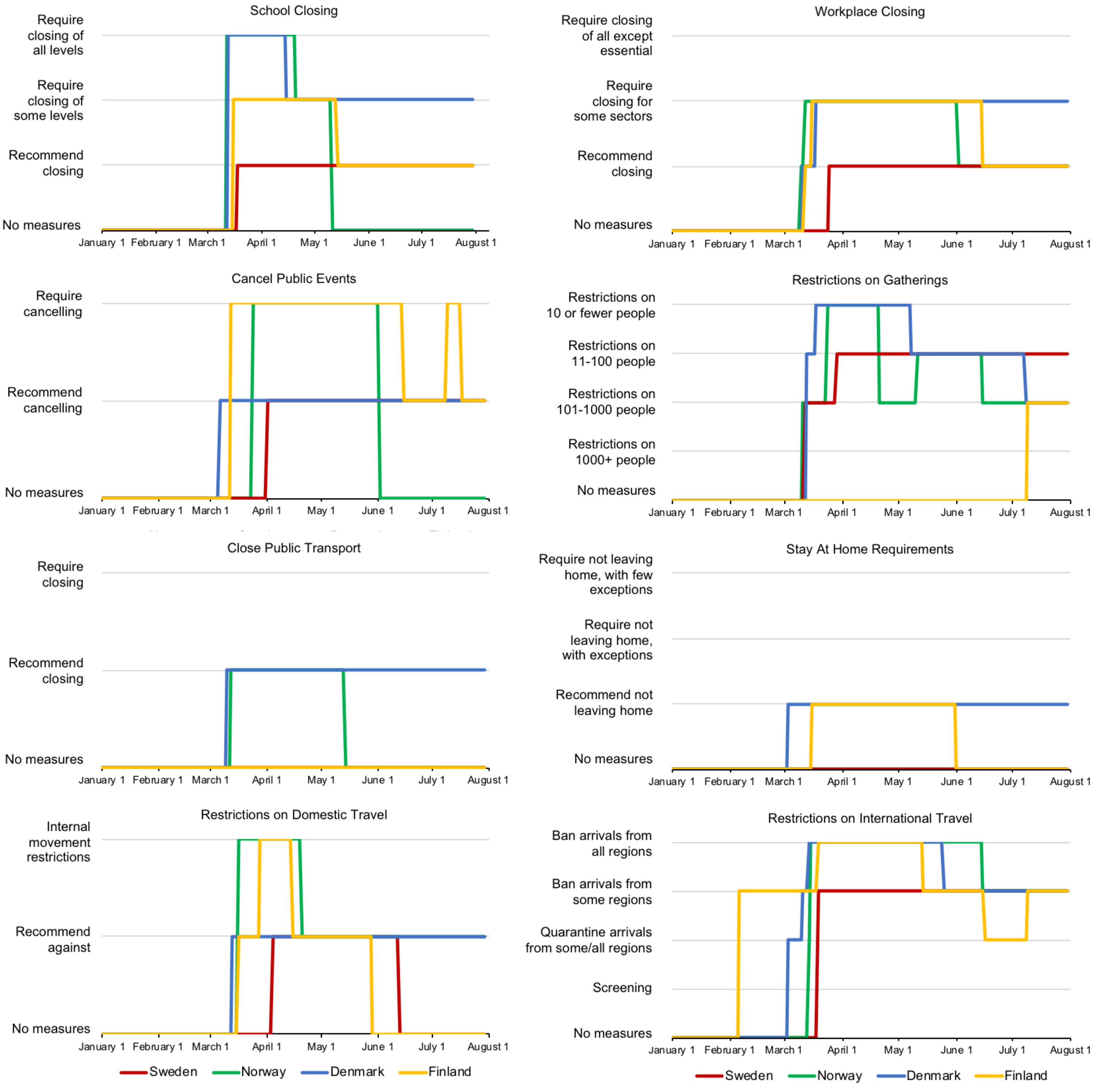

Supplementary Figure 2: A. Daily new COVID-19 cases per 1000 population. B. Daily COVID-19 tests per 1000 population. C. Daily share of COVID-19 tests that are positive. All lines represent a seven day moving average. Data is included until July 31.

A.

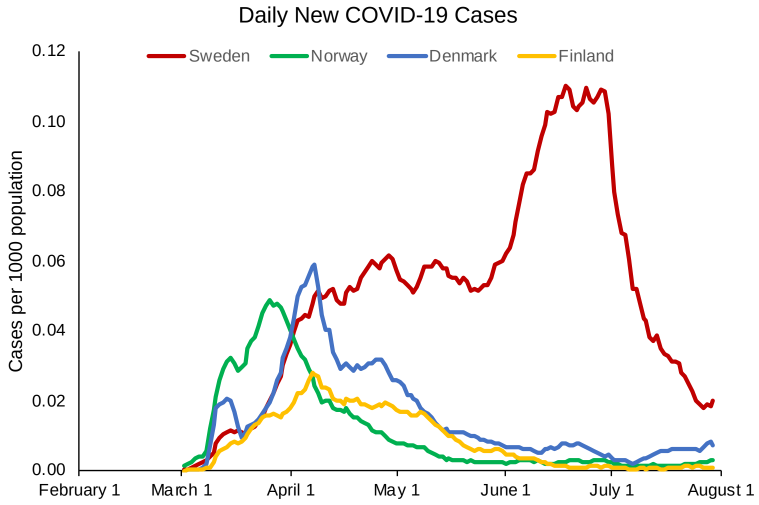

B.

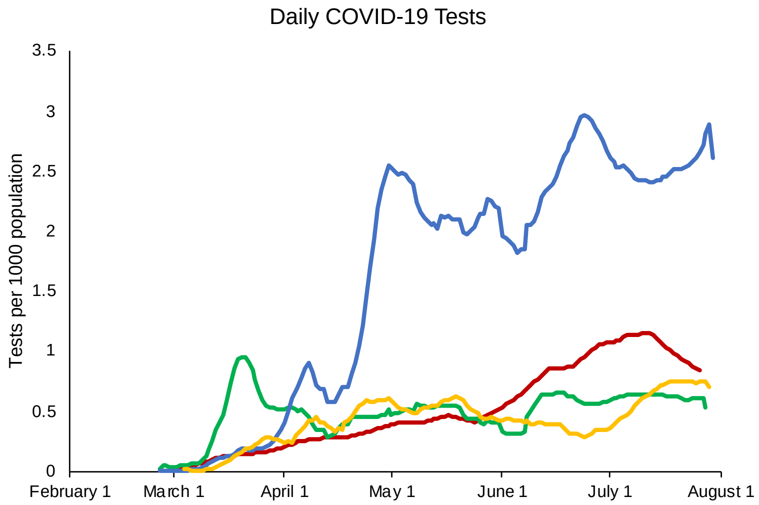

C.

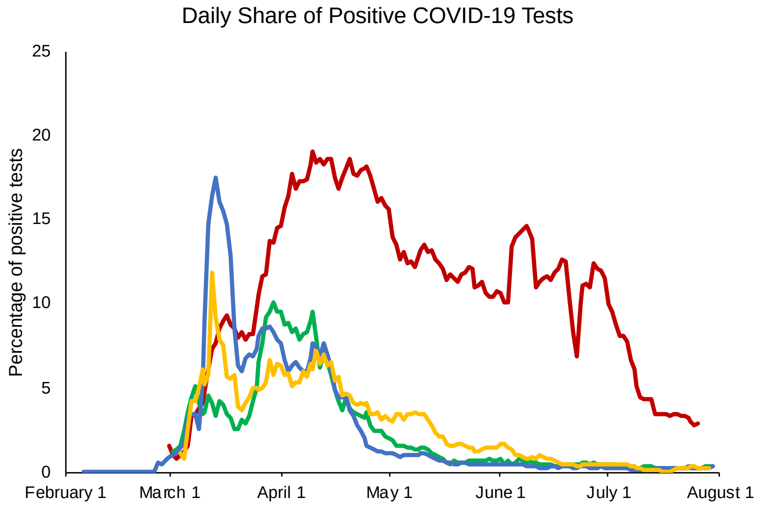

Supplementary Figure 3: A. Workplace mobility, percent change from baseline. B. Transit stations mobility, percent change from baseline. C. Parks mobility, percent change from baseline. D. Grocery and pharmacy mobility, percent change from baseline. All lines represent a seven day moving average. Baseline values are region-specific and are established using a median of the corresponding day of the week from the period between January 3 to February 6, 2020. Note: Public holidays occurred for all four countries on April 10, April 13, and May 21; on May 1 for Norway/Sweden/Finland; on June 19 for Sweden/Finland; on April 9 and June 1 for Norway/Denmark; and May 8, May 22, and June 5 for Denmark. Data is included until July 31 and is from the Google Community Mobility Report.

A.

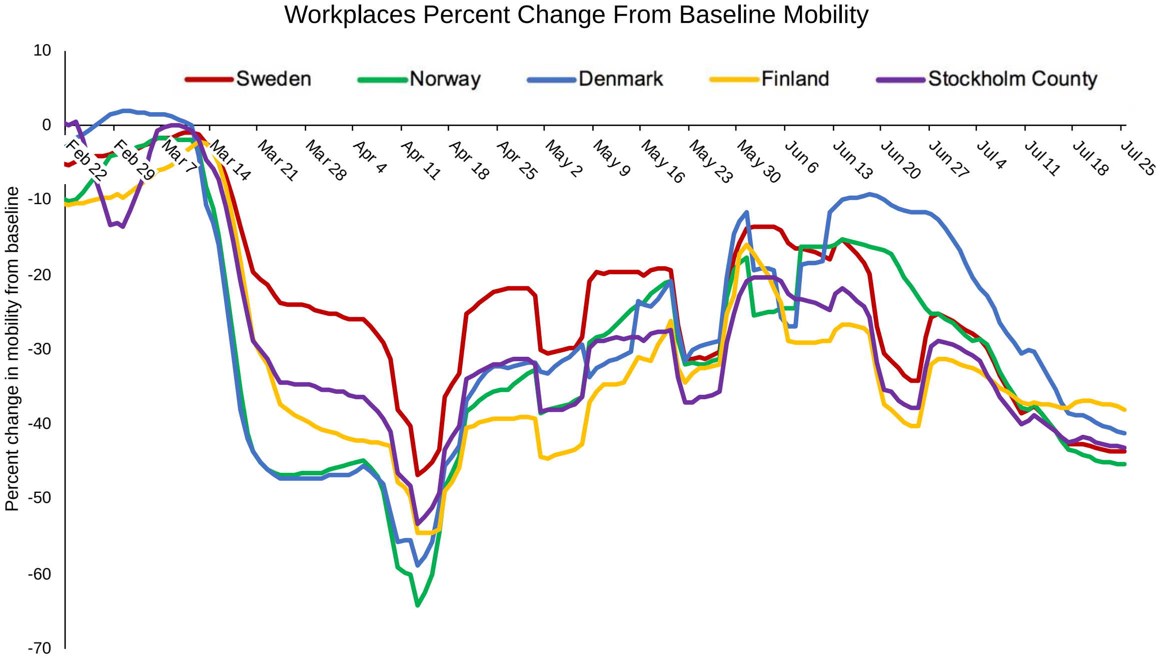

B.

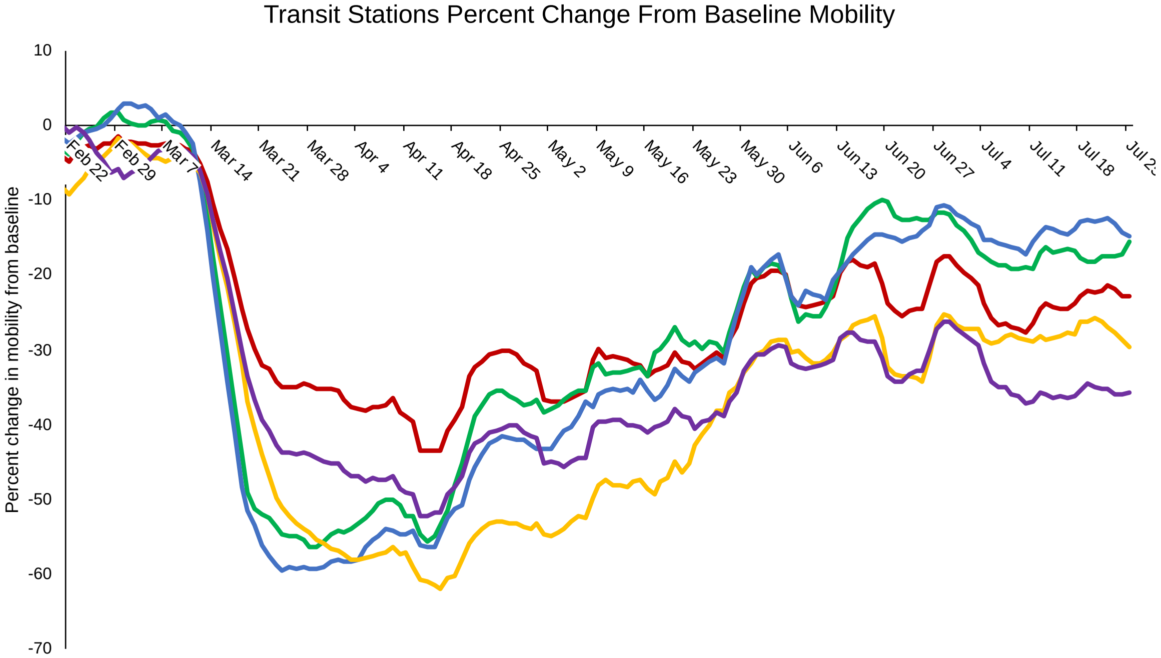

C

.

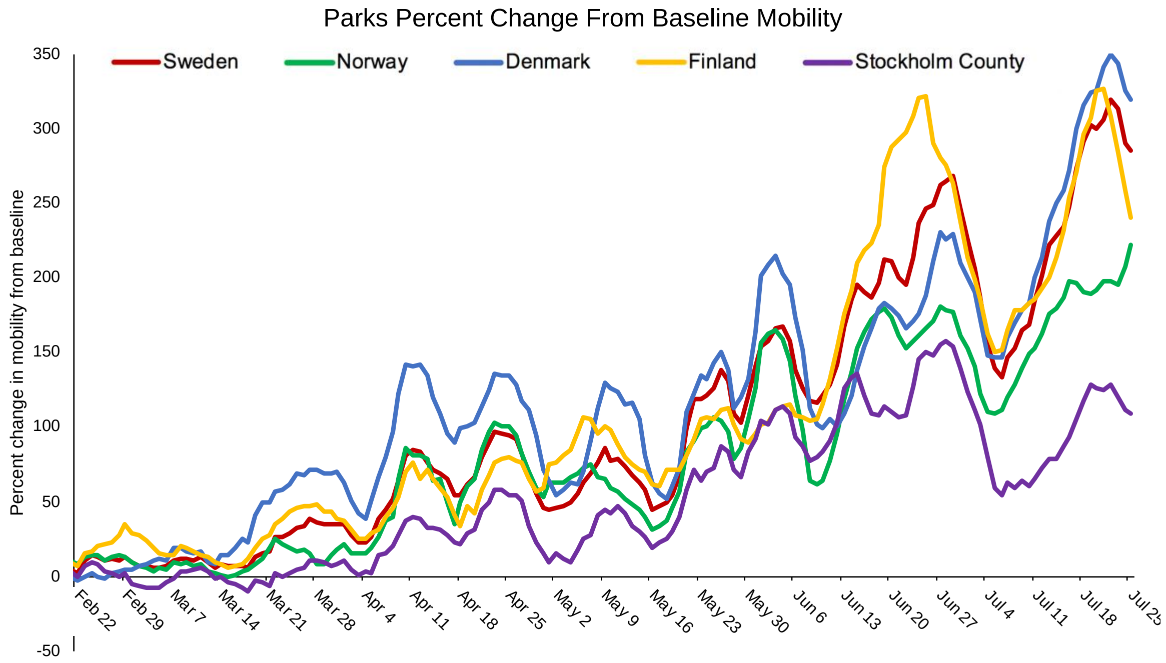
Parks Percent Change From Baseline Mobility
Sweden
Norway
Denmark
Finland
Stockholm County
Percent change in mobility from baseline
350
300
250
200
150
100
50
0
-50
Feb 22
Feb 29
Mar 7
Mar 14
Mar 21
Mar 28
Apr 4
Apr 11
Apr 18
Apr 25
May 2
May 9
May 16
May 23
May 30
Jun 6
Jun 13
Jun 20
Jun 27
Jul 4
Jul 11
Jul 18

D

.

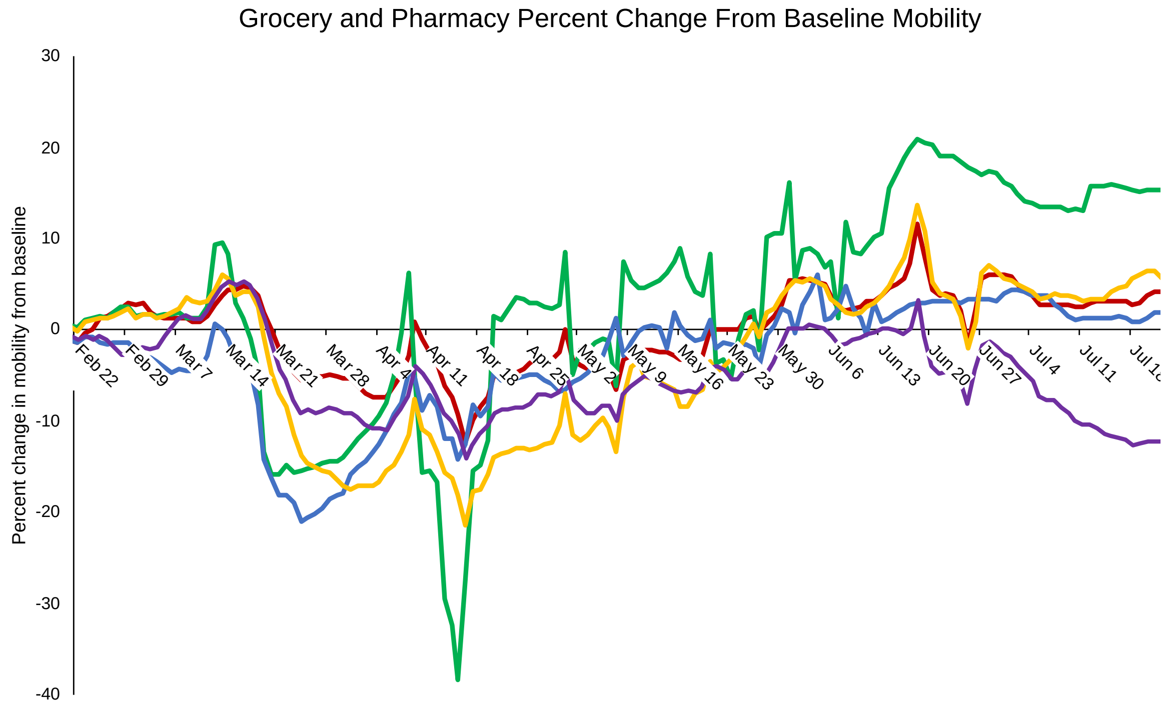
Grocery and Pharmacy Percent Change From Baseline Mobility
Percent change in mobility from baseline
30
20
10
0
-10
-20
-30
-40
Feb 22
Feb 29
Mar 7
Mar 14
Mar 21
Mar 28
Apr 11
Apr 18
Apr 25
May 9
May 16
May 23
May 30
Jun 6
Jun 13
Jun 20
Jun 27
Jul 4
Jul 11

Supplementary Figure 4: Cumulative COVID-19 deaths per 1000 population and cumulative deaths in long term care per 1000 population in Sweden, Norway, Denmark, and Finland compared to OECD average. Data from Nordic countries is from the week of July 15; OECD average data is from a report from the Canadian Institute for Health Information published on May 25. Solid bars represent deaths per 1000 population, and open bars represent deaths in long term care per 1000 population.

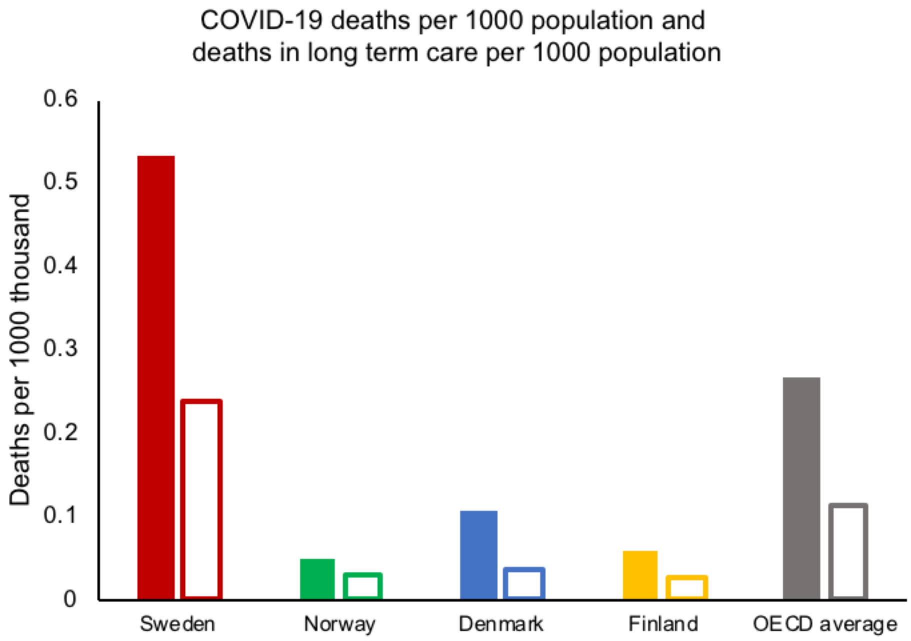

Supplementary Figure 5: Population, population density, urbanization, population in capital city, and population density in capital city of Sweden, Norway, Denmark, and Finland.

	**Sweden**	**Norway**	**Denmark**	**Finland**
Population (2020)	10,345,449	5,367,580	5,824,857	5,528,737
Population density (people/km^2)	25.4	14.7	138.7	18.2
Urbanization (%)	87.1	82.6	88.0	85.5
Population in capital city (%)	9%	13%	11%	12%
Population density in capital city (people/km^2)	5189	5244	7165	3059
